# Anatomy, Descriptive and Topographical

**Published:** 1887-10

**Authors:** 


ANATOMY DESCRIPTIVE AND TOPOGRAPHICAL IN 625 ILLUSTRATIONS. By Carl Heitzmann, M. D. English edition by Louis Heitzmann, M. D. New York: J. H. Vail & Co. 1887.

This is a most complete atlas of anatomy, each page presenting one or more illustrations with descriptive notes. It will prove a most valuable adjunct to the student of anatomy, either when used in connection with Gray or in the dissecting-room. The illustrations are beautifully executed, most of them being from drawings by the author. It cannot be too highly commended to students and practitioners. H. P. C.

